# An asparagine/glycine switch governs product specificity of human N-terminal methyltransferase NTMT2

**DOI:** 10.1038/s42003-018-0196-2

**Published:** 2018-11-02

**Authors:** Cheng Dong, Guangping Dong, Li Li, Licheng Zhu, Wolfram Tempel, Yanli Liu, Rong Huang, Jinrong Min

**Affiliations:** 1 Structural Genomics Consortium, University of Toronto, Toronto, M5G1L7 ON Canada; 2 Department of Medicinal Chemistry and Molecular Pharmacology, Center for Cancer Research, Institute for Drug Discovery, Purdue University, West Lafayette, IN 47907 USA; 3 School of Life Sciences, Jinggangshan University, 343009 Ji’an, Jiangxi China; 4 Department of Physiology, University of Toronto, Toronto, M5S 1A8 ON Canada

## Abstract

α-N-terminal methylation of proteins is an important post-translational modification that is catalyzed by two different N-terminal methyltransferases, namely NTMT1 and NTMT2. Previous studies have suggested that NTMT1 is a tri-methyltransferase, whereas NTMT2 is a mono-methyltransferase. Here, we report the first crystal structures, to our knowledge, of NTMT2 in binary complex with S-adenosyl-L-methionine as well as in ternary complex with S-adenosyl-L-homocysteine and a substrate peptide. Our structural observations combined with biochemical studies reveal that NTMT2 is also able to di-/tri-methylate the GPKRIA peptide and di-methylate the PPKRIA peptide, otherwise it is predominantly a mono-methyltransferase. The residue N89 of NTMT2 serves as a gatekeeper residue that regulates the binding of unmethylated versus monomethylated substrate peptide. Structural comparison of NTMT1 and NTMT2 prompts us to design a N89G mutant of NTMT2 that can profoundly alter its catalytic activities and product specificities.

## Abstract (web summary)

Cheng Dong et al. resolve the crystal structure of NTMT2, presenting the molecular basis for substrate recognition. Using structural and biochemical studies, they identified a specific residue within NTMT2 that controls its binding affinity to unmethylated or monomethylated substrates.

## Introduction

Protein methylation participates in regulation of a broad spectrum of cellular processes. Besides the extensively studied protein lysine/arginine methylation^1,2^, the addition of a methyl group at the free α-N-termini of proteins represents a unique mode of post-translational modification and remains underexplored, though its discovery dates back to 1976^3^. α-N-terminal methylation is conserved from prokaryotes to humans, and a variety of N-terminally methylated proteins have been identified in ribosomal and histone proteins^4,5^. Recent studies have shed some light on the functions of N-terminal methylation. For instance, in yeast, loss of N-terminal methylation of the ribosomal protein Rpt1 leads to impaired cell growth and hypersensitivity to stress^6^. In Drosophila, α-N-terminal methylation level of histone H2B increases during development^7^. In human, loss of N-terminal methylation of regulator of chromatin condensation 1 (RCC1) diminishes its binding affinity for DNA, and results in defects of spindle assembly and chromosome segregation^8^. The absence of N-terminal methylation of DNA damage-binding protein 2 (DDB2) decreases the localization of DDB2 to UV-induced DNA damage foci and hinders nucleotide excision repair^9^. The N-terminal methylation of CENP-B enhances its binding to centromeric DNA in cells^10^. The N-terminal methylation of CENP-A is not only required for cell survival, recruitment of CENP-T/I, and proper chromosome segregation, but may also accelerate tumorigenesis in p53-deficient background^11^.

The first α-N-terminal methyltransferase (NTMT), human NTMT1 (also known as METTL11A/NRMT1) and its yeast ortholog, had just been functionally characterized in 2010^5,12^, although the first crystal structure of NTMT1 had been determined in 2005 (PDB ID: 2EX4). NTMT1 is an S-adenosyl-l-methionine (SAM)-dependent methyltransferase. During the enzymatic reaction, NTMT1 transfers a methyl group from SAM to the α-amino group of the protein substrates, resulting in the production of S-adenosyl-l-homocysteine (SAH) and α-N-methylated proteins. NTMT1 recognizes proteins bearing an N-terminal X-P-K/R consensus sequence, including RCC1, RB1, DDB2, CENP-A/B, PARP3, etc.^9–15^. Knockdown of NTMT1 results in hypersensitivity of breast cancer cell lines to double-stranded DNA breaks (DSBs) and increased proliferation of estrogen receptor positive breast cancer cells MCF-7 and LCC9^16^; NTMT1 knockout mice are phenotypically defective in DNA repair and exhibit premature aging^17^. In 2013, another human NTMT, NTMT2/METTL11B/NRMT2, was described as a mono-methyltransferase^18^, although NTMT1 is able to catalyze tri-methylation^12,19,20^. We and another group previously solved crystal structures of NTMT1 in ternary complex with SAH and substrate peptides, and proposed a catalytic mechanism^13,21^. However, the molecular mechanism of methylation by NTMT2 remains elusive.

In order to unravel the molecular basis of the substrate and product specificity of NTMT2, we determine the X-ray crystal structures of NTMT2 in binary complex with SAM, as well as in ternary complex with SAH and an RCC1-derived peptide (SPKRIA). We also perform mutational analysis and comprehensively investigate the substrate specificity and product methylation states of NTMT1 and NTMT2 for a panel of 20 peptides. Our results manifest that NTMT2 is not a sole mono-methyltransferase, but is also able to fully methylate both GPKRIA and PPKRIA peptides. Furthermore, we identify N89 as a key residue for product specificity of NTMT2. The N89G mutant of NTMT2 is more active than the wild type NTMT2, which is able to convert S/APKRIA peptides from the mono-methylation state to di-/tri-methylation states in our in vitro enzymatic assays.

## Results

### Overall structure of NTMT2

**Fig. 1:**
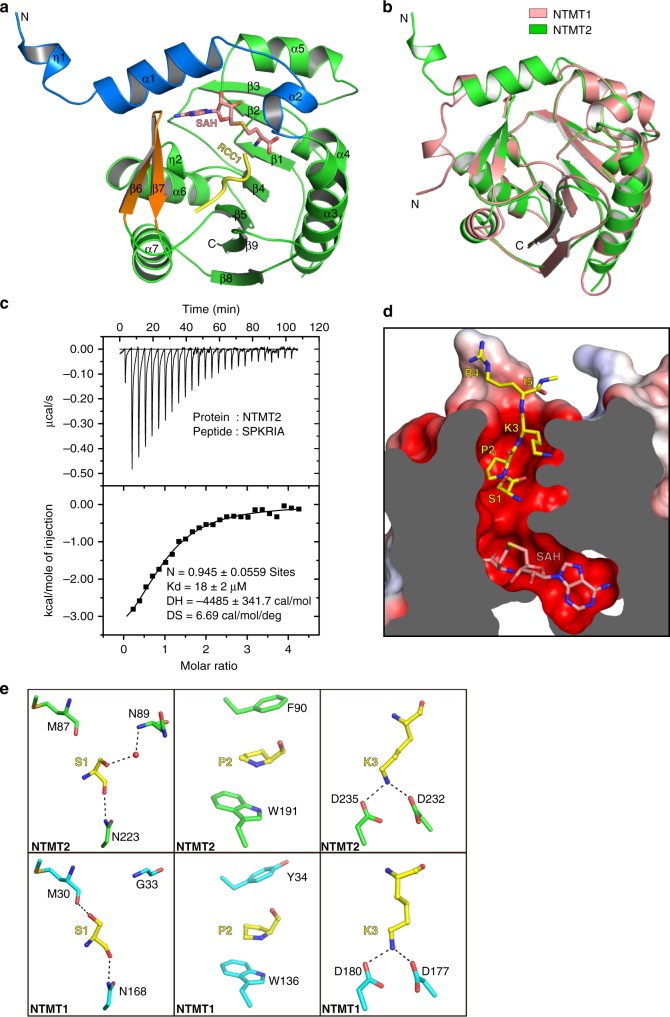
Overall structure of NTMT2. **a** Ribbon diagram of NTMT2 in ternary complex with SAH and a SPKRIA peptide derived from N-terminal RCC1. **b** Structural alignment of NTMT1 (PDB: 5E1B) and NTMT2. **c** Isothermal titration calorimetry measurement of the interaction between NTMT2 and the SPKRIA peptide. **d** Cross-section view of the SAH-SPKRIA-binding pocket. The electrostatic potential surface of NTMT2 plotted at ±5 kT/e (red, negative; blue, positive). **e** Close-up views of the S-P-K motif recognition by NTMT2 and NTMT1 (PDB: 5E1B). Residues of NTMT2 and NTMT1 involved in the interactions are labeled and shown in green and cyan, respectively. Hydrogen bonds are indicated as dashed lines, and water molecule is shown as red sphere

So far, two NTMTs that methylate X-P-K/R are identified in mammals. NTMT1 has been classified as a tri-methyltransferase^12,19,20^, whereas NTMT2 was reported as a mono-methyltransferase^18^. To determine the molecular basis of different product specificities between NTMT2 and NTMT1, we solved crystal structures of the NTMT2-SAM binary complex and the NTMT2-SAH-SPKRIA peptide  ternary complex. Despite that an unmethylated peptide was used for crystallization, a methylated α-amino group in the N-terminus of the peptide was traced in the crystal structure. Crystal diffraction data and model refinement statistics are summarized in Table 1. Based on the folding pattern, NTMT2 is a SAM-dependent class I methyltransferase^22^, which consists of a central seven-stranded β sheet (β1–β5 and β8–β9), flanked by three α-helices (α3–α5) and two α-helices (α6–α7) on each side, respectively (Fig. 1a). In addition, NTMT2 contains two auxiliary regions: an N-terminal α-lid (η1, α1–α2), and a β-lid (β6–β7) inserted between β5 and α8. These two lids cover the core domain that contributes to the substrate recognition (Fig. 1a, Supplementary Fig. 1a). The overall architecture of NTMT2 closely resembles that of NTMT1 with a backbone RMSD below 1 Å (Fig. 1b).

**Table 1:** Data collection and refinement statistics

	NTMT2-SAM	NTMT2-SAH-SPKRIA
*Data collection*		
Space group	*P* 2_1_ 2_1_ 2	*P* 4_3_
Cell dimensions		
* a*, *b*, *c* (Å)	45.59, 132.96, 42.29	44.36, 44.36, 262.06
* α*, *β*, *γ* (°)	90.00, 90.00, 90.00	90.00, 90.00, 90.00
Resolution (Å)	45.60-2.00 (2.05–2.00)	31.14–1.20 (1.22–1.20)
*R*_sym_ or *R*_merge_	0.086 (0.700)	0.074 (0.973)
*I*/*σI*	10.2 (1.7)	11.0 (2.0)
Completeness (%)	94.4 (91.7)	96.0 (93.2)
Redundancy	2.6 (2.6)	6.1 (6.6)
*Refinement*		
Resolution (Å)	18.98–2.00	31.10–1.20
No. of reflections	16,002	145,394
*R*_work_/*R*_free_	0.208/0.250	0.154/0.178
No. of atoms	1780	4160
Protein	1691	3690
Ligand/ion	–	72
Water	63	314
*B*-factors	27.4	17.0
Protein	27.3	16.1
Ligand/ion	–	17.6
Water	28.9	25.7
R.m.s. deviations		
Bond lengths (Å)	0.014	0.02
Bond angles (°)	1.7	1.9

The values in parentheses are for highest-resolution shell

The NTMT2-SAM binary complex subtly differs from the NTMT2-SAH-SPKRIA ternary complex in that the turn of the β-lid of the ternary complex is shifted closer to the catalytic core, which facilitates hydrogen bonding between substrate K3 and both D232 and D235 of NTMT2 (Supplementary Fig. 1b). The co-factor SAH/SAM in the ternary complex is nearly superimposable with that in the substrate-free binary complex. Based on sequence alignments, the N-terminal methyltransferases have a conserved DxGxGxGR motif located on the β1–α4 loop, which is involved in the SAH/SAM binding (Supplementary Fig. 1c). Indeed, the carboxylate moiety of SAH forms a salt bridge with R129, and its amino group is held by hydrogen bonds with the G124 and Q190 carbonyls, respectively (Supplementary Fig. 1d). Its ribosyl moiety is stacked against Y76 with its 2′,3′-diol group coordinated by D146. Its adenine group forms hydrogen bonds with the Q175 side-chain carboxamide and the L174 main-chain amide group, respectively. M147, corresponding to NTMT1 I92, forms a methionine–π interaction with the adenine group as well (Supplementary Fig. 1d). As a result, the SAH molecule is deeply buried by the α-lid and the substrate is inserted into the conserved binding pocket (Supplementary Fig. 2a).

### NTMT1 and NTMT2 employ a similar substrate recognition mode

RCC1 is a physiological substrate of NTMT1^12^, but no physiological substrates have been reported for NTMT2. To compare substrate recognition by NTMT2 and NTMT1, we first examined whether NTMT2 could bind to any known NTMT1 substrates like RCC1. Isothermal titration calorimetry (ITC) shows that NTMT2 is able to bind to the N-terminal hexapeptide of RCC1 (SPKRIA) with a *K*_d_ value of 18 μM (Fig. 1c), which is ~20-fold weaker than that of NTMT1 (*K*_d_ value of 0.8 μM)^13^. Our ternary complex structure shows that, like in NTMT1, the substrate peptide is deeply inserted into the binding pocket through hydrophilic and hydrophobic interactions, and the α-amine of the first residue (S1) points towards the sulfur atom of SAH (Fig. 1d). In the NTMT2 complex, S1 fits snugly into the channel, where the main-chain O atom is anchored by N223 in NTMT2 (N168 in NTMT1) through hydrogen bonding (Fig. 1e, Supplementary 2b). The N223A mutant abrogates substrate binding affinity and has no enzymatic activity (Supplementary Fig. 2c, d). The side chain of S1 has adopted the p conformation^23^ and interacts, mediated by solvent, with the main chain of N89 in NTMT2, whereas in the NTMT1 complex, S1 present in the m conformer hydrogen bonds with the NTMT1 main chain at M87 (Fig. 1e).

The substrate residue P2 is sandwiched by the aromatic side chains of W191 and F90 (Fig. 1e). W191 also appears to play an important role in NTMT2 structural stability, as mutations of W191 to A or even hydrophobic amino acids Y, L or I, resulted in insoluble proteins. The ε-amine of substrate K3 interacts with two aspartates in both NTMT1 and NTMT2 structures (Fig. 1e). Neither mutant D232A nor D235A of NTMT2 exhibits any measurable interaction with the substrate or the enzymatic activity (Supplementary Fig. 2c, e, f). In NTMT2, the main-chain N and O atoms of K3 are involved in a water-mediated hydrogen bond with the side chain of N223 and a direct hydrogen bond with the main chain of I270, respectively (Supplementary Fig. 2b).

Substrate residues downstream of the S-P-K motif protrude from the pocket on the surface of NTMT2 (Fig. 1d), indicating that they are not essential for the recognition. Indeed, the fourth residue R4 is not involved in direct interaction and the main chain of the fifth residue I5 is accommodated by Q268 (Supplementary Fig. 2b). In summary, aside from minor differences, the sets of residues responsible for substrate binding in NTMT1 and NTMT2 overlap (Fig. 1e), which explains why these two enzymes share an X-P-K/R substrate recognition motif.

### NTMT2 also acts as a di-/tri-methyltransferase

**Fig. 2:**
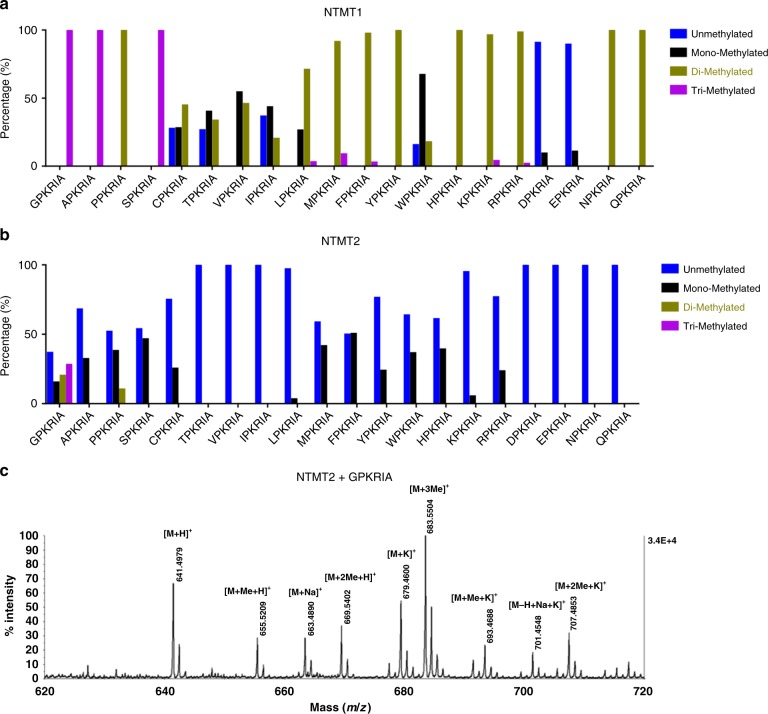
NTMT1 and NTMT2 exhibit different methylation patterns. MALDI-MS analysis of 20 peptides catalyzed by NTMT1 (**a**) and NTMT2 (**b**). Synthetic peptides of XPKRIA with the first position (X) being any of the 20 standard amino acids are used in this study. **c** MALDI-MS analysis of GPKRIA peptide catalyzed by NTMT2

Previous studies have indicated that NTMT1 can catalyze mono-methylation, di-methylation, and tri-methylation, but NTMT2 was reported as a mono-methyltransferase^12,18–20^. Comparison of the substrate-binding sites (Fig. 1e) does not provide a straightforward explanation. In order to gain a full understanding of the methylation states of the products, we synthesized 20 XPKRIA peptides with X being any of the 20 standard amino acids, and characterized the methylation progression of these peptides by both NTMT1 and NTMT2 using matrix-assisted laser desorption/ionization mass spectrometry (MALDI-MS) technique under the same conditions^19^ (Fig. 2a, b, Supplementary Data 1). Since the enzymatic activity of NTMT2 is much lower than that of NTMT1, we had to use higher concentration of NTMT2 (2 μM) in our assay. We also increased the NTMT1 concentration from previously used 0.2 to 2 μM^13^ for comparison. Our results revealed that NTMT1 could exhaustively methylate [A/G/P/S]-PKRIA peptides to full degree of methylation states, and mainly di-methylate the other peptides. Of note, NTMT1 even methylated [D/E]-PKRIA, albeit the catalytic activities for those two peptides were very low (Fig. 2a), as we expected that the negatively charged pocket would repel these substrates^13^. Compared with NTMT1, NTMT2 has a weaker catalytic capability, which only mono-methylated most XPKRIA peptides. Surprisingly, NTMT2 could convert GPKRIA peptide into mono-methylation, di-methylation, and tri-methylation states (Fig. 2b, c). Moreover, NTMT2 was able to di-methylate the PPKRIA peptide despite low yield (Fig. 2b). Taken together, NTMT2 is more than a mono-methyltransferase, but also functions as di-/tri-methyltransferase, depending on the identity of the first amino acid of the substrates.

### N89 of NTMT2 serves as a gatekeeper for the catalytic activity and alters its product specificity

NTMT2, unlike NTMT1, failed to di-/tri-methylate SPKRIA (Fig. 2a, b). Comparison of the substrate-binding sites in NTMT1 (M30, N168, W136, D177, and D180) and NTMT2 (M87, N223, W191, D232, and D235) revealed that the residues that engaged the direct substrate binding were identical (Fig. 1e). Hence, these direct substrate-binding residues are not responsible for such different methylation states of products generated by NTMT1 and NTMT2.

**Fig. 3:**
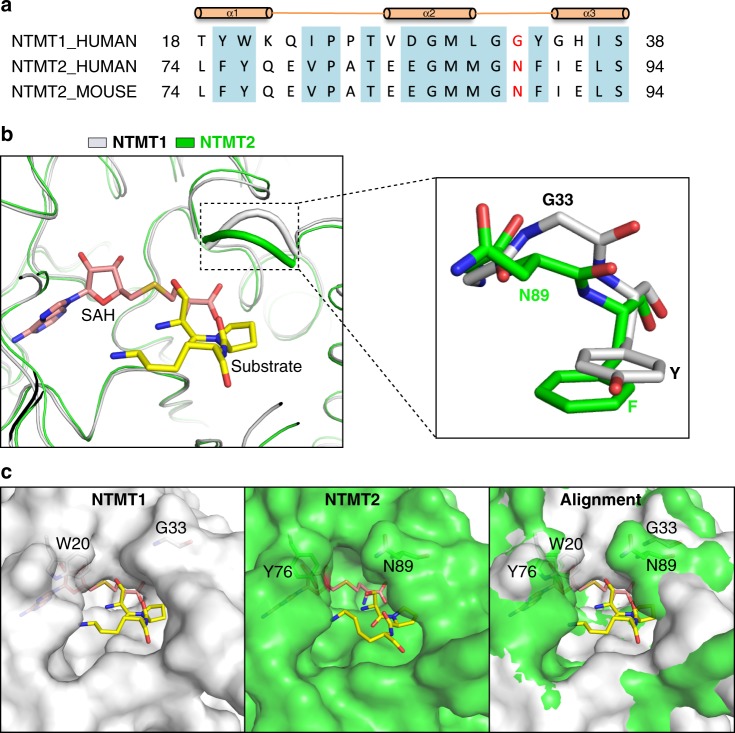
N89 of NTMT2 serves as a gatekeeper residue to regulate the substrate entry into the active pocket. **a** Sequence alignment of human NTMT1, human NTMT2, and mouse NTMT2. The G33 of NTMT1 and N89 of NTMT2 are colored in red. **b** Structural alignment of NTMT1-SPKRIA (PDB: 5E1B) (gray) and NTMT2-SPKRIA (green) complexes. The Ω loops are indicated by dashed lines and the residues of the Ω loops are labeled. The Ω loop of NTMT1 adopts an open conformation, whereas NTMT2 acts as a relatively closed conformation. **c** Surface representations of the substrate active pocket from NTMT1 (gray) and NTMT2 (green). The Y76 of NTMT2 generates a large empty space above the S atom of SAH compared to W20 of NTMT1. The N89 of NTMT2 creates a narrow substrate-binding channel compared to G33 of NTMT1

In order to understand the molecular basis for product specificities between NTMT1 and NTMT2, we compared the residues near the substrate-binding sites and found that two tandem aromatic residues near the α-amino group of the peptide are different between NTMT1 and NTMT2 (FY75-76 in NTMT2 and YW19-20 in NTMT1) (Fig. 3a, Supplementary Fig. 3a, b). Previous studies have shown that the aromatic residue mutant Y305F of SET7/9, or Y334F of SET8 could alter the specificity of the SET domain histone lysine methyltransferases from a mono-methyltransferase to a di-methyltransferase^24–26^. Therefore, we investigated both single and double mutants of NTMT2 including F75Y, Y76W, and FY75-76YW, but none of them altered the methylation state of NTMT2 (Supplementary Fig. 3c–e), which is consistent with a recent report^27^. One reason is that the Y76 of NTMT2 or Y19 of NTMT1 is 5.4 and 6.8 Å away from the α-amino group, respectively (Supplementary Fig. 3a, b). Such long distances lack the ability to form a direct CH···O hydrogen bond with a methylated α-amino group as observed in the SET domain methyltransferases^26^. Nevertheless, the wild-type NTMT2 can carry out tri-methylation of G-PKRIA peptide and di-methylation of P-PKRIA peptide, indicating that α-amino groups of some substrates are able to be further deprotonated and fully methylated by NTMT2. Therefore, we hypothesized that other residue(s) near the substrate-binding site may regulate such product specificity.

**Fig. 4:**
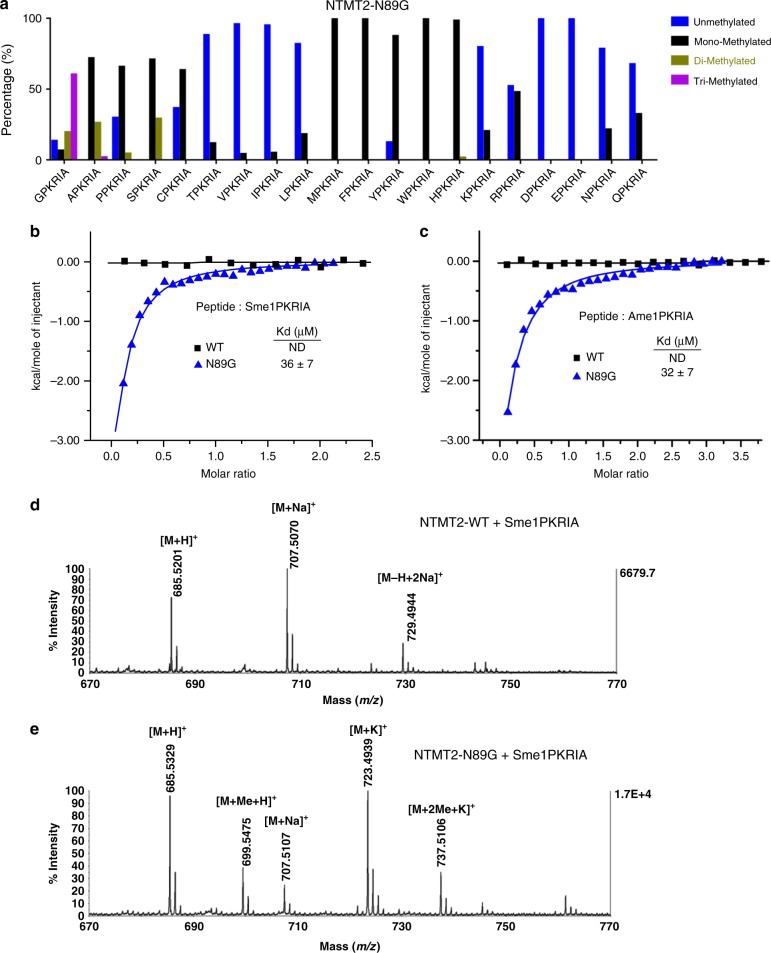
The NTMT2 N89G mutant exhibits increased methylation activity. **a** MALDI-MS analysis of 20X-PKRIA peptides catalyzed by NTMT2-N89G mutant. ITC measurements of the interactions between the wild-type (WT) or N89G mutant NTMT2 and the Sme1PKRIA (**b**) or Ame1PKRIA peptide (**c**). ND indicates no detectable binding. **d** MALDI-MS analysis of Sme1PKRIA peptide catalyzed by the WT NTMT2. **e** MALDI-MS analysis of Sme1PKRIA peptide catalyzed by the NTMT2 N89G mutant

We then scrutinized the overall structures of NTMT1 and NTMT2 and identified a small conformational change in the α2–α3 loop, referred to as Ω loop here. NTMT1 adopts an open conformation, while NTMT2 displays a relatively closed conformation (Fig. 3b). Close examination identified that NTMT1 carries a G33, whereas NTMT2 harbors an N89 at the same location (Fig. 3a). The comparison of the surface representation of NTMT2 and NTMT1 indicates that the N89 in NTMT2 creates a narrower substrate-binding channel compared to G33 in NTMT1 (Fig. 3c). Based on this observation, we generated the NTMT2 N89G mutant and analyzed the methylation products by the MALDI-MS assay. Our results reveal that N89G mutant display enhanced activity than wild-type NTMT2, and has a higher rate of mono-methylation (Fig. 4a, Supplementary Data 1). Remarkably, it completely methylates the SPKRIA peptide to mono-methylation and di-methylation states. Moreover, it also efficiently converts the APKRIA peptide to mono-methylation and di-methylation states, as well as to a tri-methylation state (Fig. 4a). Therefore, N89 functions as a gatekeeper for the substrate binding and product specificity of NTMT2.

### The N89G mutant has a higher affinity for mono-methylated peptide than wild type

We hypothesized that the long side chain of N89 in NTMT2 might cause steric hindrance for binding the methylated SPKRIA (Sme1PKRIA) peptide. Indeed, wild-type NTMT2 barely demonstrated any binding affinity, while the single point mutation of N89G in NTMT2 bound to Sme1PKRIA peptide with a *K*_d_ value of 36 μM (Fig. 4b). Likewise, wild-type NTMT2 had no detectable binding to Ame1PKRIA, but the N89G mutant exhibited a *K*_d_ value of 32 μM towards Ame1PKRIA (Fig. 4c). Consistent with these binding data, our MALDI-MS results showed that wild-type NTMT2 did not produce any di-methylation or tri-methylation products (Fig. 4d), but N89G mutant has robust enzymatic activity towards Sme1PKRIA (Fig. 4e). These observations also explain why NTMT2 is only capable of transferring one methyl group to the α-amine of serine, whereas N89G could further methylate mono-methylated serine. Taken together, the smallest glycine, even when it was mono-methylated or di-methylated (Gme1/2-PKRIA) could still insert into the binding pocket. But the mono-methylation of any amino acid other than glycine or proline would result in products that were not able to pass through the gatekeeper N89 and thereby would be detached from the NTMT2 catalytic site. Nevertheless, the N89G mutant would expand the substrate-binding channel and facilitate the substrates access to the active site, resulting in enhanced enzymatic activity, even alteration of the product specificity from mono-methylation to tri-methylation.

### Proposed catalytic mechanism of NTMT2

Although there is neither direct hydrogen bond formed between NTMT2 and the substrate α-amino group, nor a general base that is responsible for deprotonating α-amino group, there are multiple water molecules present at the active channel that mediated the interaction between α-amino group of substrates and the active site (Supplementary Fig. 4a). We proposed that the α-amino group of the first residue is further stabilized by multiple water molecules-mediated hydrogen bonds, orienting the acceptor α-amino group toward the sulfonium ion of SAM in close proximity. Y76, H195, D222, and D235 serve as general bases for proton abstraction from the substrate α-amino group through multiple water-mediated hydrogen-bonding networks (Supplementary Fig. 4b, c). The deprotonated α-amine is a better nucleophile to attack the methyl group of SAM to accomplish the methyl transfer.

## Discussion

We determined the crystal structure of NTMT2 in ternary complex with cofactor SAH and a substrate peptide SPKIRA, which is consistent with its classification as a SAM-dependent class I methyltransferase^22^. It has been proposed that NTMT2 acts as a mono-methyltransferase^18^. In this study, we comprehensively investigated the substrate recognitions of NTMT1 and NTMT2, and the methylation state specificities of their products. To our knowledge, this is the first study to indicate that NTMT2 can also achieve tri-methylation or di-methylation when the glycine or proline is present at first residue of substrates, respectively. Our data also suggest that NTMT2 employs an S_N_2 nucleophilic attack mechanism by multiple water-mediated deprotonation of α-amino group. Like NTMT1, NTMT2 processes the methylation reaction by a dynamic distributive mechanism (Fig. 2c), in which NTMT2 releases the product after each methyl addition, and then rebind for further methylation^20^. Notably, NTMT2 does not bind to Sme1PKRIA or Ame1PKRIA peptide in vitro, resulting in loss of the di-methylation activity. Although both human N-terminal methyltransferases share a high degree of similarity in terms of global structures, NTMT2’s product specificity and enzymatic activity clearly distinguishes itself as a unique cellular methyltransferase. We found that the Ω loop of NTMT2 guards the substrate-binding pocket in a relatively stricter manner compared to that of NTMT1. We also identify that N89 serves as a gatekeeper residue to control the entry of substrate into the active pocket. Accordingly, we generated a N89G mutant of NTMT2 that exhibited higher enzymatic efficiency towards the substrates. In addition, this mutant is sufficient to bind to Ame1PKRIA or Sme1PKRIA peptide, and converts them to tri-methylation or di-methylation, respectively. NTMT1 and NTMT2 exhibit different expression patterns. For instance, the NTMT1 mRNA is abundantly expressed in mouse brain and ubiquitously expressed in human tissues, whereas the NTMT2 mRNA is strongly expressed in mouse and human muscles^18,28^. Therefore, NTMT1 and NTMT2 may contribute to different biological activities in a cellular context-dependent manner. After the initiator methionine excision, the proteins bearing a G-P-K/R consensus sequence at the N-terminus (for instance, CENP-A/B) could be converted to tri-methylation states by both NTMT1 and NTMT2. For the other types of substrates, NTMT2 primarily carries out mono-methylation, while NTMT1 is responsible for further di-/tri-methylation.

## Methods

### Protein expression and purification

DNA encoding human NTMT2 (residues 58–278) was amplified by PCR and cloned into pET28-MKH8SUMO vector using the InFusion^TM^ cloning kit (ClonTech) employing the manufacturer’s instructions. This recombinant plasmid contains an SUMO tag and TEV cleavage site at the N-terminus. The protein was overexpressed in *Escherichia coli* BL21 (DE3) competent cells after induction with 0.2 mM Isopropyl β-d-1-thiogalactopyranoside (IPTG) at 16 °C overnight. Cells were lysed in 20 mM Tris–HCl pH 7.5, 400 mM NaCl, 5% glycerol and 2 mM beta-mercaptoethanol buffer and purified by Ni-NTA agarose chromatography. The SUMO tag was cleaved by TEV protease at 4 °C overnight and removed by reloading onto the Ni-NTA. The protein was diluted and applied onto HiTrap Q HP anion exchange chromatography column (GE Healthcare) equilibrated with 20 mM Tris–HCl pH 7.5, 25 mM NaCl and 0.5 mM tris (2-carboxyethyl) phosphine (TCEP). The proteins were eluted with a linear gradient of 0–50% elution buffer (20 mM Tris–HCl pH 7.5, 1 M NaCl and 0.5 mM TCEP). The proteins were further purified by gel filtration Superdex 200 10/300 (GE Healthcare). The gel filtration buffer contains 20 mM Tris–HCl pH 7.5, 150 mM NaCl and 0.5 mM TCEP. The purified protein was concentrated to 20 mg mL^−1^ for crystallization. Mutants were created by QuickChange PrimeSTAR Mutagenesis Basal Kit and verified by DNA sequencing, the primers used in this study are listed in Supplementary Table 1. Mutant proteins were purified with the same protocol as wild type. The expression and purification of NTMT1 were performed as described previously^13^. Briefly, the corresponding gene encoding NTMT1 (residues 2–223) was subcloned into pET28a-LIC expression vector and then expressed at 16 °C overnight in the Terrific Broth medium. The protein was purified by Ni-NTA affinity column. The eluted protein was further purified by gel filtration Superdex 200 10/300 (GE Healthcare) pre-equilibrated with 20 mM Tris–HCl pH 7.5, 150 mM NaCl and 0.5 mM TCEP.

### Crystallization and structure determination

Since NTMT2 purified from *E. coli* contains endogenous methyl donor SAM/SAH, so we did not add any additional SAM/SAH during the crystallization. The NTMT2–SAM complex was crystallized in 20% (w/v) PEG3350 and 0.2 M sodium acetate via sitting drop vapor diffusion by mixing 1 μL protein and 1 μL reservoir solution at 4 ℃. To get the ternary complex crystal, the protein was incubated with SPKRIA peptide (from 100 mM stock) at a molar ratio of 1:1.5 for 1 h on ice before setting up the crystallization trial. The crystals of NTMT2 in complex with SPKRIA were obtained in 30% PEG2000 (w/v) and 0.1 M potassium thiocyanate at 18 °C. The crystals were cryo-protected in the reservoir solution supplemented with 20% (v/v) glycerol and flash-frozen in liquid nitrogen.

Diffraction data were collected under cooling in a stream of cold nitrogen gas. Symmetry-related reflection intensities were merged with AIMLESS^29^ software. The program PHASER^30^ was used for molecular replacement. Atomic models of the crystal structures were interactively rebuilt with COOT. Model geometry was validated with PHENIX.MOLPROBITY^31^. Details for individual crystal structures follow.

Diffraction data for the ternary NTMT2–SAH–SPKRIA complex were collected first on a rotating copper anode source and processed with XDS^32^. The structure was solved by molecular replacement with coordinates from PDB entry 5E1D^13^ and automatically rebuilt with ARP/wARP^33^. Further restrained refinement in REFMAC5^34^ and interactive rebuilding of the model were performed against another data set that was collected at the Canadian Light Source beamline 08ID^35^ and processed with HKL-3000^36^. Atomic anisotropic displacement parameters were analyzed on the PAVARTI^37^ server.

Diffraction data for the SAM–NTMT2 complex were collected on a rotating copper anode and processed with XDS. Coordinates from the ternary complex model were used for molecular replacement. Restrained refinement of the atomic model was performed with REFMAC5 and BUSTER^38^.

### Isothermal titration calorimetry

ITC measurements were performed in 20 mM Tris–HCl pH 7.5, 150 mM NaCl and 0.5 mM TCEP at 25 °C using MicroCal VP-ITC instrument. The final concentrations of protein and peptide were 50–80 μM and 0.6–1.2 mM, respectively. The peptide was titrated into the protein solution with 26 injections of 10 μL each. Injections were spaced 180 s with a reference power of 15 μcal s^−1^. The ITC data were processed using Origin software.

### Methyltransferase activity assays

A mixture (36 μL) of 2 μM enzyme (NTMT1 or NTMT2), 25 mM Tris–HCl pH 7.5, 50 mM KCl and 200 μM SAM was incubated at 37 °C for 5 min. 4 μL of 100 μM peptide substrate (final concentration 10 μM) was then added to initiate the reaction. The reaction was incubated overnight and then stopped with quenching solution (20 mM NH_4_H_2_PO_4_, 0.4% (v/v) TFA in 1:1 acetonitrile/water). Reaction mixtures were analyzed with an Applied Biosystems Voyager matrix-assisted laser desorption/ionization time-of-flight mass spectrometer. Processing of all spectra results in Data Explorer included application of a noise filter (correlation factor of 1.0) and a baseline correction. Areas of the monoisotopic peaks for all relevant species were combined to obtain a total area for each sample. The fraction of each methylation state was calculated by summing the areas of all the monoisotopic peaks for that state ([M + H]^+^, [M + Na]^+^, [M + K]^+^, [M – H + 2Na]^+^, [M – H + 2 K]^+^, and [M – H + Na + K]^+^) and then dividing by the total area.

## Electronic supplementary material

Supplementary information

Description of Supplementary Data 1

Supplementary Data 1

## Data Availability

The datasets generated during the current study are available from the corresponding author on reasonable request. The atomic coordinates and structure factors have been deposited in the RCSB Protein Data Bank. The accession codes for the NTMT2-SAM and NTMT2-SAH-SPKRIA are 5UBB and 6DUB, respectively.
