# Supplementary material for: An asparagine/glycine switch governs product specificity of human N-terminal methyltransferase NTMT2
Source: Commun Biol. 2018 Nov 2;1:183. doi: 10.1038/s42003-018-0196-2 (PMC6214909; doi:10.1038/s42003-018-0196-2)
Supplement: Supplementary file 1 — Supplementary information [file 42003_2018_196_MOESM1_ESM.pdf]

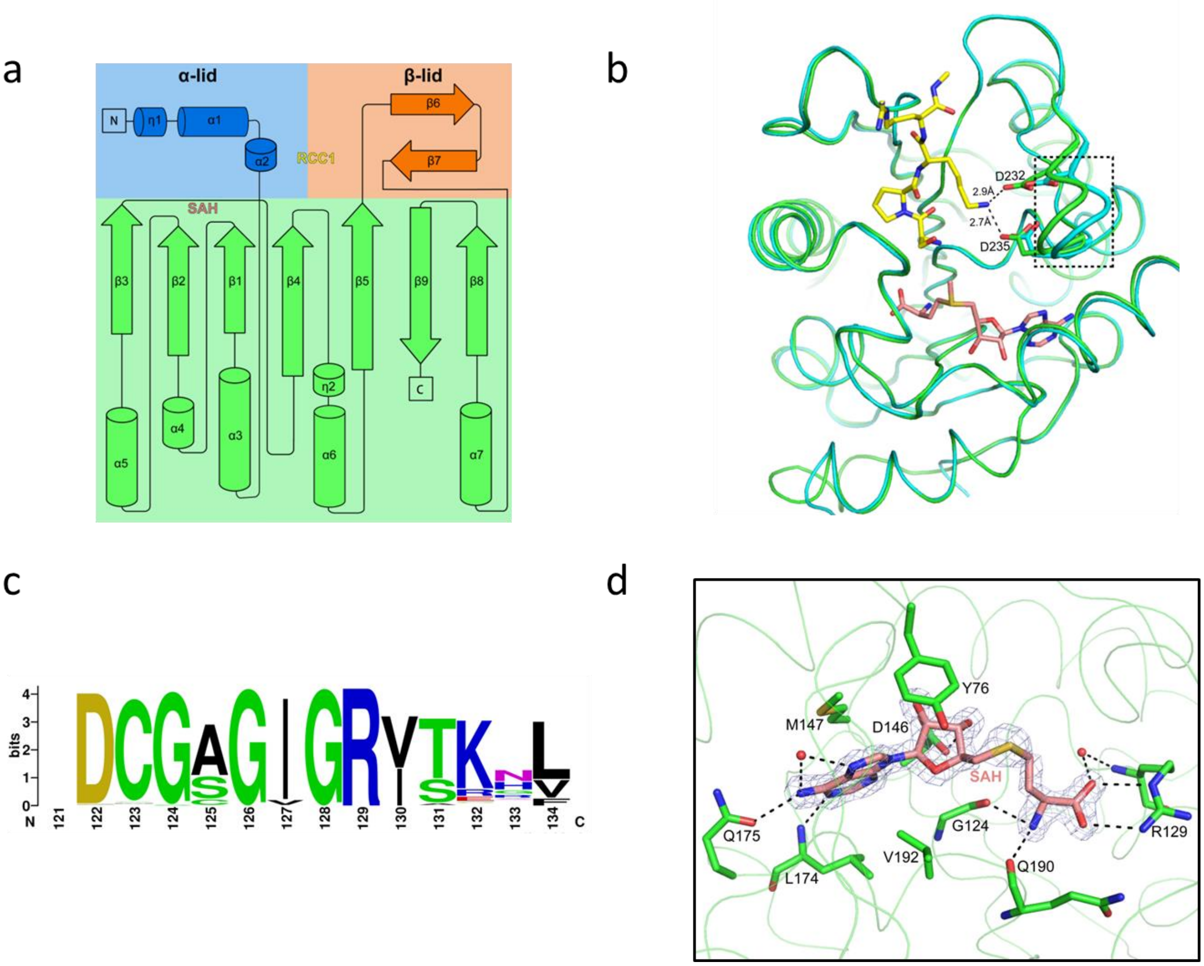

Supplementary Figure 1: a, Topology diagram of NTMT2. **b**, Structure comparison of NTMT2 substrate-free (cyan) and NTMT2-SPKRIA form (green). The β-lid loop (β6-β7) is indicated in dashed lines and D232 and D235 residues are shown as sticks. **c**, WebLogo[1] of the conserved consensus motifs in the SAH binding site of NTMT2, the sequences are selected from 150 homologs by the ConSurf server[2]. **d**, Close-up view of the interactions of NTMT2 with co-factor SAH. Residues of NTMT2 involved in the interactions are labeled and shown in green. SAH is shown in salmon. 2Fo-Fc electron density map of SAH contoured at 2 σ level. The water molecules are shown as red spheres. Hydrogen bonds are indicated as dashed lines.

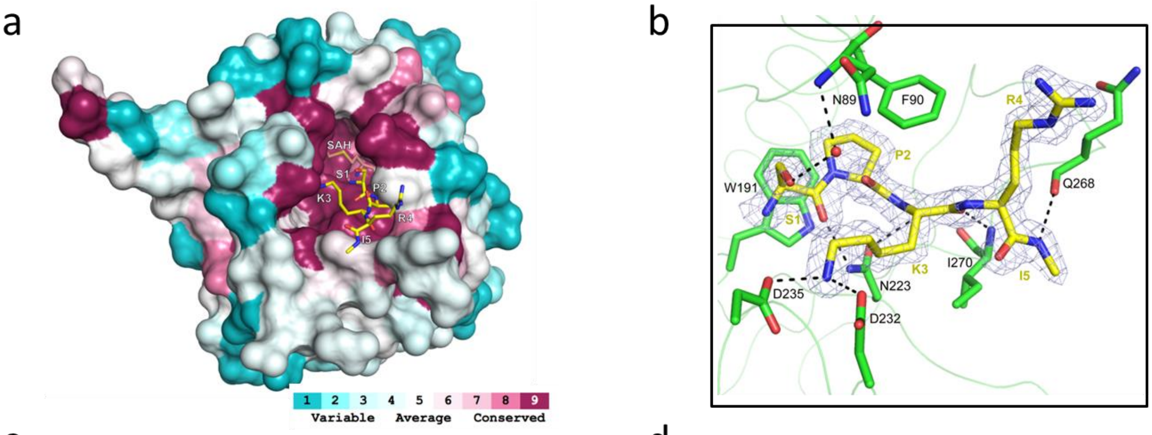

c

Enzyme	ITC/K_d (μM)
NTMT2-N223A	ND
NTMT2-D232A	ND
NTMT2-D235A	ND

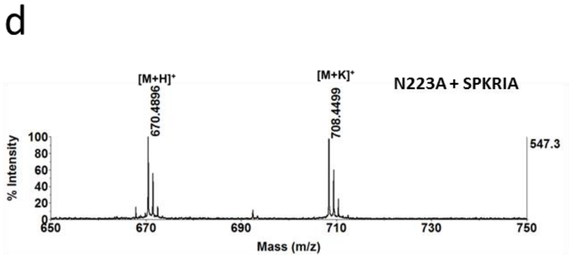

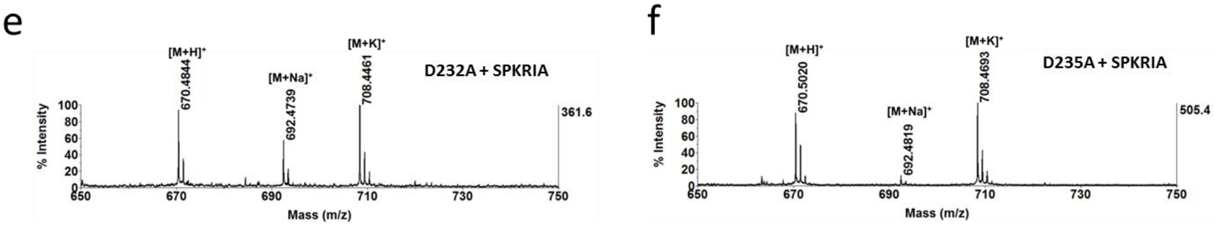

Supplementary Figure 2: a, Conservation analysis of NTMT2 using the ConSurf server[2]. The residues of NTMT2 are colored based on their conservation grades using the color-coding bar, with turquoise-through-maroon indicating variable-through-conserved. The SAH and peptide SPKRI is shown as salmon and yellow stick, respectively. **b**, Close-up view of the interactions of NTMT2 with SPKRIA peptide. Residues of NTMT2 involved in the interactions are labeled and shown in green. Water molecule is shown as red sphere. 2Fo-Fc electron density map of SPKRIA peptide contoured at 2 σ level. Hydrogen bonds are indicated as dashed lines. **c**, ITC measurements of the binding affinities of NTMT2 and mutants with SPKRIA peptide. ND indicates no detectable binding. (**d-f**) Catalytic activity analysis of the NTMT2 mutants with SPKRIA peptide by MALDI-MS.

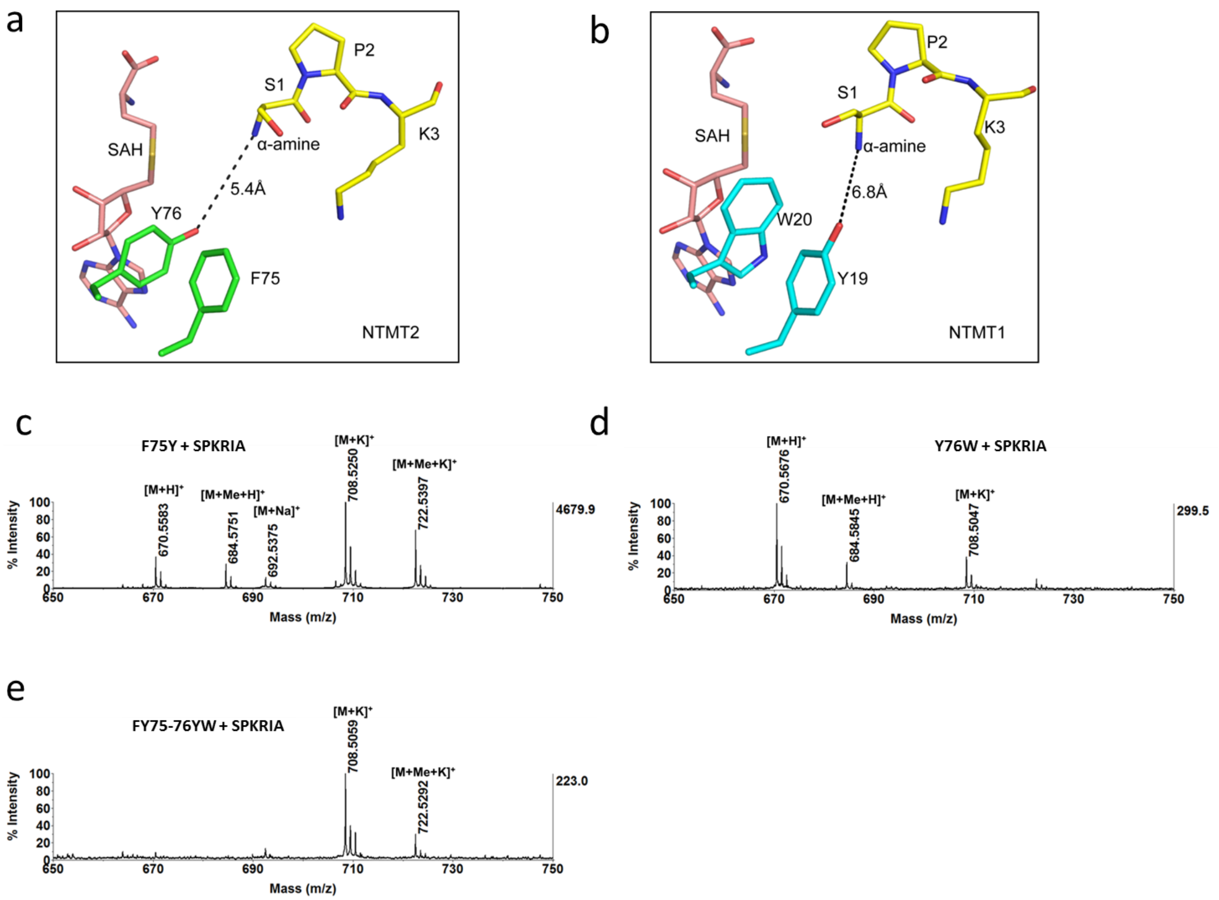

Supplementary Figure 3: Close-up views of the aromatic residues near the active site of NTMT2-SPKRIA (**a**) and NTMT1-SPKRIA (**b**) (PDB: 5E1B) The distances are indicated as dashed lines. (**c-e**) Catalytic activity analysis of NTMT2 mutants with SPKRIA peptide by MALDI-MS.

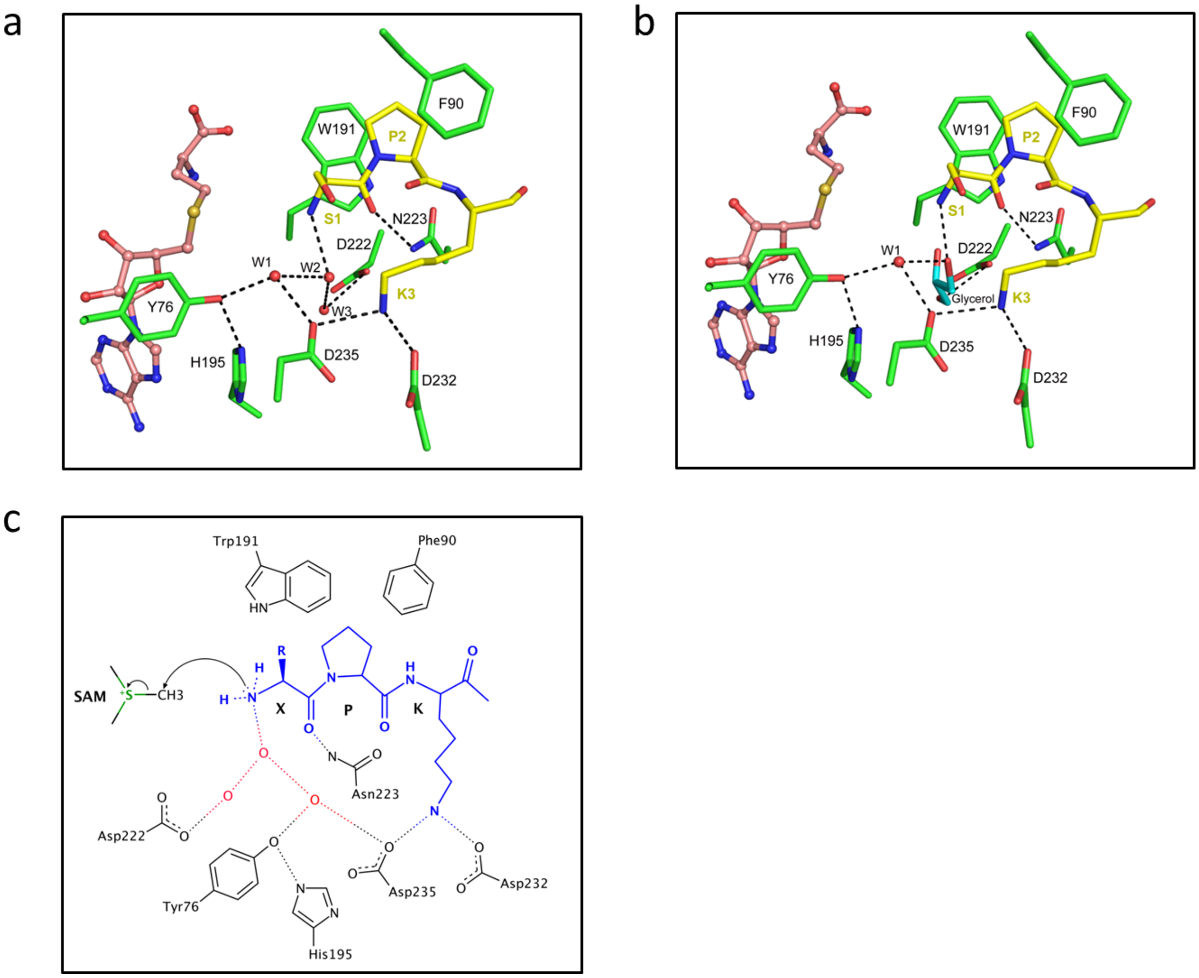

Supplementary Figure 4: Catalytic mechanism of NTMT2. **a**, SAH and substrate is shown as stick in salmon and yellow, respectively. Putative catalytic water molecules are shown as red spheres. In the crystallographic models, corresponding positions of W2 and W3 are occupied by glycerol (cyan) (**b**). **c**, Schematic illustration of the proposed catalytic mechanism of X-P-K motif by NTMT2.

Supplementary Table 1 List of primers used in this study

Name	Forward primer	Reverse primer
NTMT2(58-278)	TTGTATTTCCAGGGCACGTCCCAGGTTATT	CAAGCTTCGTCATCAGTGCAGGGCGAACAT
F75Y	AAGCTTTATTATCAAGAGGTCCCAGCA	TTGATAATAAAGCTTGGCCCGCGCGTA
Y76W	CTTTTTTGGCAAGAGGTCCCAGCAACT	CTCTTGCCAAAAAAGCTTGGCCCGCGC
FY75-76YW	AAGCTTTATTGGCAAGAGGTCCCAGCAACT	CTCTTGCCAATAAAGCTTGGCCCGCGCGTA
N89G	ATGGGAGGTTTCATAGAGCTGTCGAGC	TATGAAACCTCCCATCATCCCTTCTTC
W191A	ATTCAGGCGGTAAGTGGGCATTTAACG	ACTTACCGCCTGAATCCATATCACGTC
W191Y	ATTCAGTACGTAAGTGGGCATTTAACG	ACTTACGTACTGAATCCATATCACGTC
W191L	ATTCAGTTAGTAAGTGGGCATTTAACG	ACTTACTAACTGAATCCATATCACGTC
W191I	ATTCAGATCGTAAGTGGGCATTTAACG	ACTTACGATCTGAATCCATATCACGTC
N223A	AAAGACGCTGTGGCGCGAGAGGGTTGT	CGCCACAGCGTCTTTCAGAATAATGAT
D232A	ATCCTCGCTTTATCCGATTCGAGCGTT	GGATAAAGCGAGGATACAACCCTCTCG
D235A	TTATCCGCTTCGAGCGTTACTCGTGAC	GCTCGAAGCGGATAAATCGAGGATACA

Supplementary References

1 Crooks, G. E., Hon, G., Chandonia, J. M. & Brenner, S. E. WebLogo: a sequence logo generator. *Genome research* **14**, 1188-1190, doi:10.1101/gr.849004 (2004).

2 Ashkenazy, H., Erez, E., Martz, E., Pupko, T. & Ben-Tal, N. ConSurf 2010: calculating evolutionary conservation in sequence and structure of proteins and nucleic acids. *Nucleic Acids Res* **38**, W529-533, doi:10.1093/nar/gkq399 (2010).
