# Supplementary material for: An asparagine/glycine switch governs product specificity of human N-terminal methyltransferase NTMT2
Source: Commun Biol. 2018 Nov 2;1:183. doi: 10.1038/s42003-018-0196-2 (PMC6214909; doi:10.1038/s42003-018-0196-2)
Supplement: Supplementary file 2 — Description of Supplementary Data 1 [file 42003_2018_196_MOESM2_ESM.docx]

**Description of Additional Supplementary Files**

**File Name**: Supplementary Data 1

**Description**: The source data underlying the graphs and charts presented in the figures (Fig. 2a, 2b, 4a) are available in Supplementary Data 1. Quantitative analysis of methylation states of 20 peptides catalyzed by NTMT1, NTMT2 and NTMT2-N89G mutant using matrix-assisted laser desorption/ionization mass spectrometry (MALDI-MS) technique under the same conditions. The first tab “NTMT1” relates to the source data underlying the graphs presented in the Fig. 2a, the second tab “NTMT2” and the third tab “NTMT2-N89G” relate to the Fig. 2b and Fig. 4a, respectively.
